# Supplementary material for: Changes to gut amino acid transporters and microbiome associated with increased E/I ratio in Chd8+/− mouse model of ASD-like behavior
Source: Nat Commun. 2022 Mar 3;13:1151. doi: 10.1038/s41467-022-28746-2 (PMC8894489; doi:10.1038/s41467-022-28746-2)
Supplement: Supplementary file 1 — Supplementary Information [file 41467_2022_28746_MOESM1_ESM.pdf]

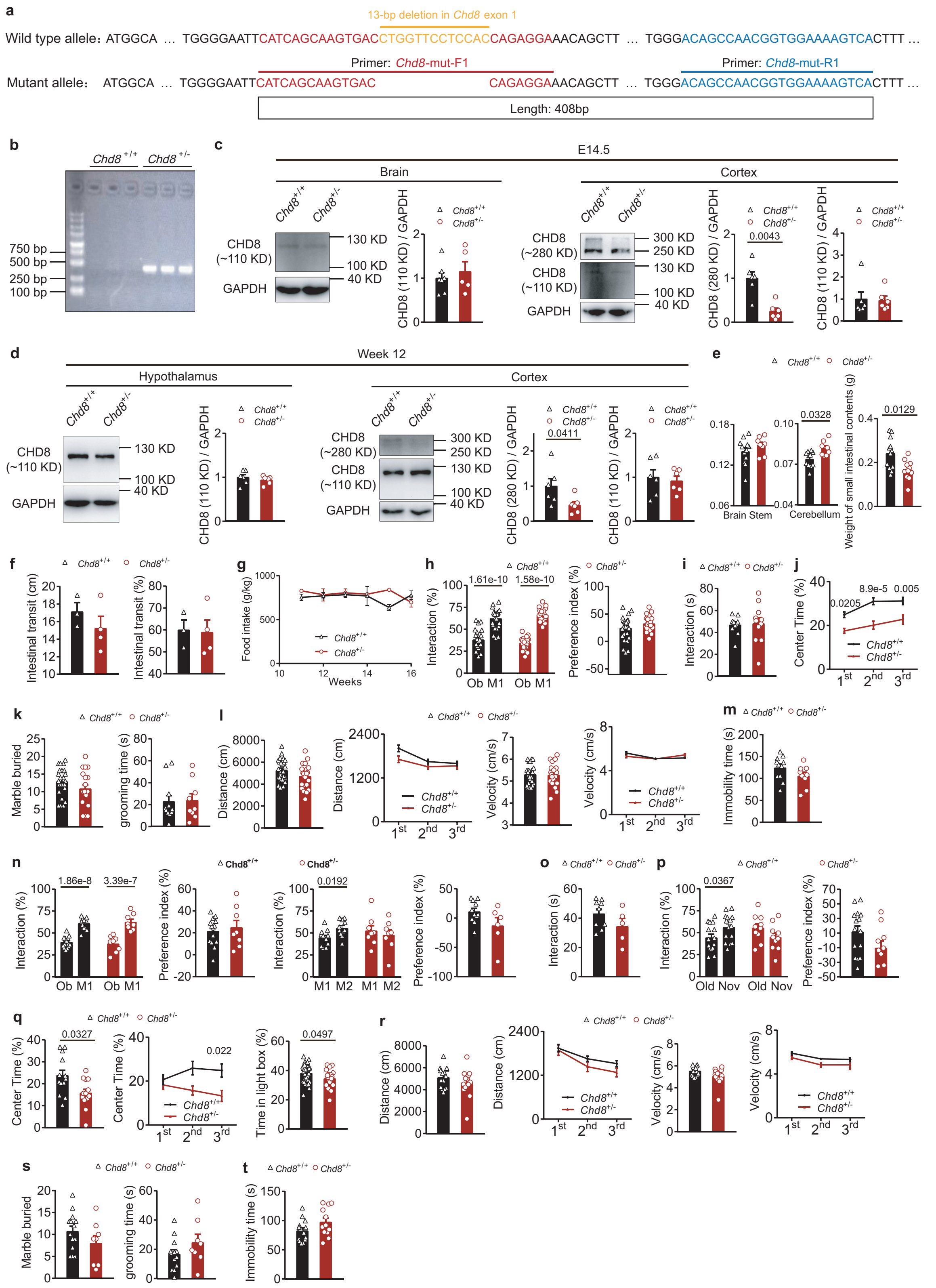

Supplementary Fig. 1 | *Chd8*$^{+/-}$ mice show abnormalities in the brain and intestine and ASD-like behaviors. a, Sequences of wild-type and mutant alleles of *Chd8* showing specific PCR primers targeting the mutant allele (*Chd8*-mut-F1/*Chd8*-mut-R1). **b**, Agarose gel electrophoresis of PCR products with specific primers targeting the mutant allele of

Chd8 (*Chd8*-mut-F1/*Chd8*-mut-R1). This experiment was repeated more than three times with similar results. **c**, CHD8 protein (110 kDa and 280 kDa) expression in the whole brain and the cerebral cortex at the embryonic stage. n = 7, 5, 6, 6, 6 and 6 mice, respectively. **d**, CHD8 protein (110 kDa and 280 kDa) expression in the hypothalamus and the cerebral cortex at week 12 (n=6 mice). **e**, Weights of the small intestinal contents of 12-week-old mice. n = 12, 8, 11, 8, 12 and 11 mice, respectively. **f**, The distance traveled by the charcoal marker (left) and the percentages of distance traveled by the charcoal marker relative to the total length of the small intestine (right) in the gastrointestinal transit test. n = 3, 4, 3 and 4 mice, respectively. **g**, The weight of food pellets consumed per kg body weight for each cage of mice. n = 2, 3, 3, 3, 3 and 3 cages, respectively. **h**, Percentages of the interaction time (left) and the preference index (right) in the social ability session of the three-chamber social interaction test. Ob, object**.** n = 24 and 20 mice, respectively. **i**, The time spent in close social interaction in the reciprocal social interaction test. n = 10 and 11 mice, respectively. **j**, Percentages of time in the center in the open-field test during the first ten minutes (1st), the second ten minutes (2nd), and the last ten minutes (3rd). n = 26 and 24 mice, respectively. **k**, Marble buried in the marble-burying test (left) and grooming time in the self-grooming test (right). n = 24, 20, 11 and 9 mice, respectively. **l**, The total distance traveled (left two panels) and the velocity of movement (right two panels) in the open-field test across the total 30 minutes and every ten minutes. n = 26 and 24 mice, respectively. **m**, The immobility time in the forced swimming test. n = 11 and 9 mice, respectively. **n**-**t**, The behavioral tests of the female mice. **n**, Percentages of the interaction time and the preference index in the social ability session (left two panels) and social novelty preference session (right two panels) of the three-chamber social interaction test. Ob, object. n = 14 and 8 mice, respectively. **o**, The time spent in close social interaction in the reciprocal social interaction test. n = 9 and 5 mice, respectively. **p**, Percentages of the interaction time (left) and the preference index (right) in the novel object recognition test. Nov, novel object; Old, old object. n = 15 and 10 mice, respectively. **q**, Percentages of time in the center in the open-field test across the total 30 minutes (left) and across every ten minutes (middle), and percentages of time spent in the light box in the light/dark box test (right). n = 13, 13, 30 and 21 mice, respectively. **r**, The total distance traveled (left two panels) and the velocity of movement (right two panels) in the open-field test across the total 30 minutes and every ten minutes. n = 15 and 13 mice, respectively. **s**, Marble buried in the marble-burying test and grooming time in the self-grooming test. n = 14, 8, 15 and 8 mice, respectively. **t**, The immobile time in the forced swimming test. n = 14 and 12 mice, respectively. Source data are provided as a Source Data file. Quantitative data are shown as the mean ± SEM. Statistical analysis was determined by the two-tailed Mann-Whitney test (**c**, **d**, **e**, **f**, **h** (right panel), **i, k**, **l** (first and third panel), **m**, **n** (second and last

panel), **o**, **p** (right panel), **q** (first and third panel), **r** (first and third panel), **s** and **t**) and two-way ANOVA with two-tailed Turkey's test for multiple comparisons (**h** (left panel), **j**, **l** (second and last panel), **n** (first and third panel), **p** (left panel), **q** (second panel) and **r** (second and last panel)). Significance was indicated by *P* value.

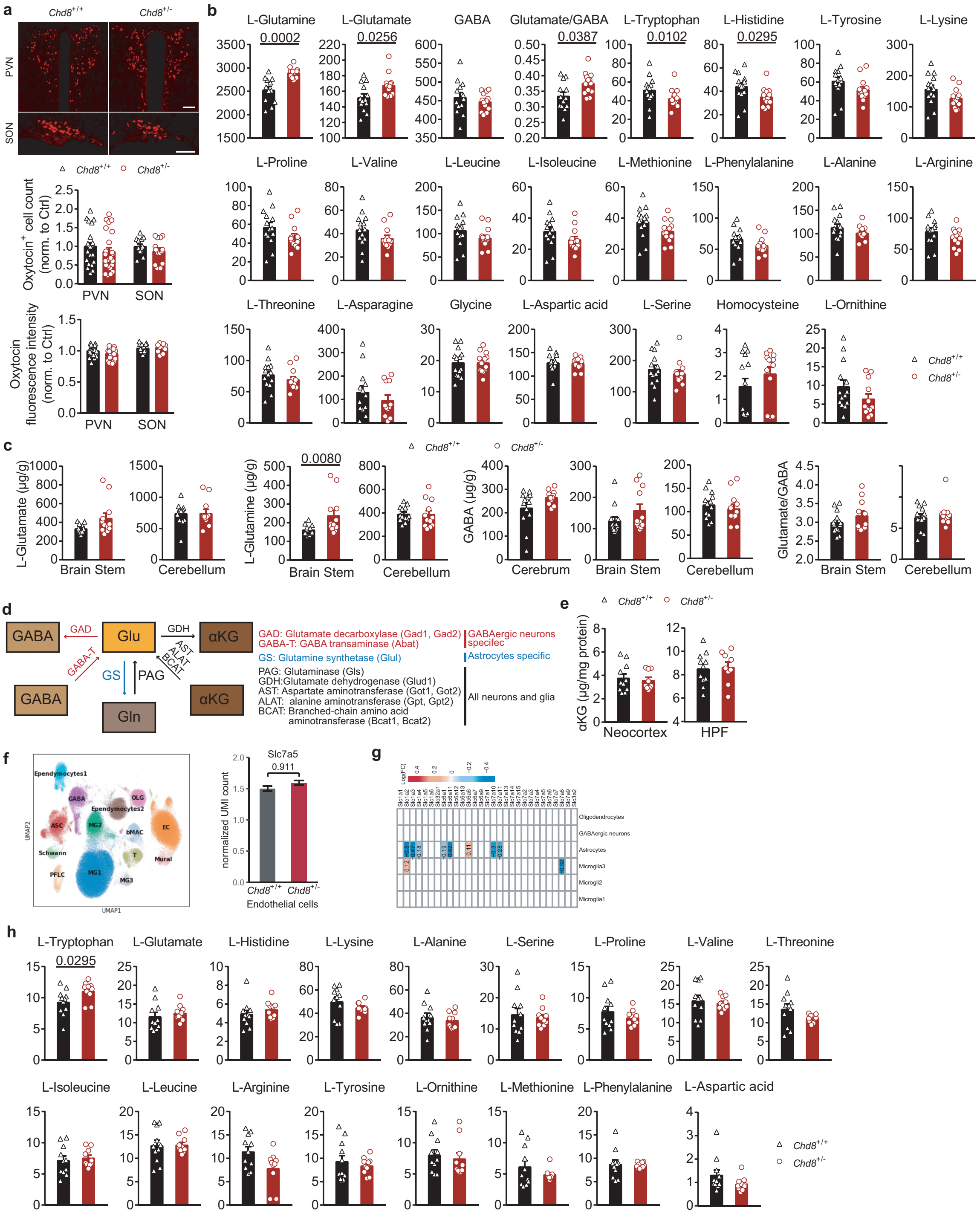

Supplementary Fig. 2 | The E/I imbalance in the *Chd8*$^{+/-}$ mice is involved in abnormalities in the brain. a, Immunofluorescence staining of oxytocin in the PVN and the SON of the mice at week 12. Scale bar, 100 μm. n=3 mice per group. Each symbol

represents one image; five to six images per mouse. n= 23, 23, 14, 14 symbols, respectively. **b**, Targeted metabolomics assay for amino acids in the whole brain of 12-week-old mice (μg/g) (n=13 mice). **c**, Levels of glutamate (μg/g), glutamine (μg/g), GABA (μg/g), and glutamate/GABA in the cerebrum, brainstem, and cerebrum of 12-week-old mice detected by targeted metabolomics assays. n = 13 and 12 mice, respectively. **d**, Schematic diagram showing the metabolism of L-glutamate in the brain. **e**, ELISA analysis of αKG in the neocortex and hippocampus (HPF) of 12-week-old mice. n = 11 and 9 mice, respectively. **f**, Single-cell profiles of brains from 12-week-old mice and gene expression of *Slc7a5* in endothelial cells from scRNA-seq data. The bar plots represented the mean of abundance of *Slc7a5* in endothelial cells (n=7665 and 8943 cells in *Chd8*$^{+/+}$ and *Chd8*$^{+/-}$ mice, respectively). Error bars represent 95% confidence interval (CI) of mean value. **g**, Fold changes of amino acid transporters in the adult brain of *Chd8*$^{+/-}$ mice from scRNA-seq. **h**, Targeted metabolomics assays for amino acids in the serum of 12-week-old mice. n = 11 and 10 mice, respectively. Source data are provided as a Source Data file. Quantitative data are shown as the mean ± SEM. Statistical analysis was determined by the two-tailed Mann-Whitney test (**b**, **c**, **e**, **f** and **h**) and two-way ANOVA with two-tailed Turkey's test for multiple comparisons (**a**). Significance was indicated by *P* value.

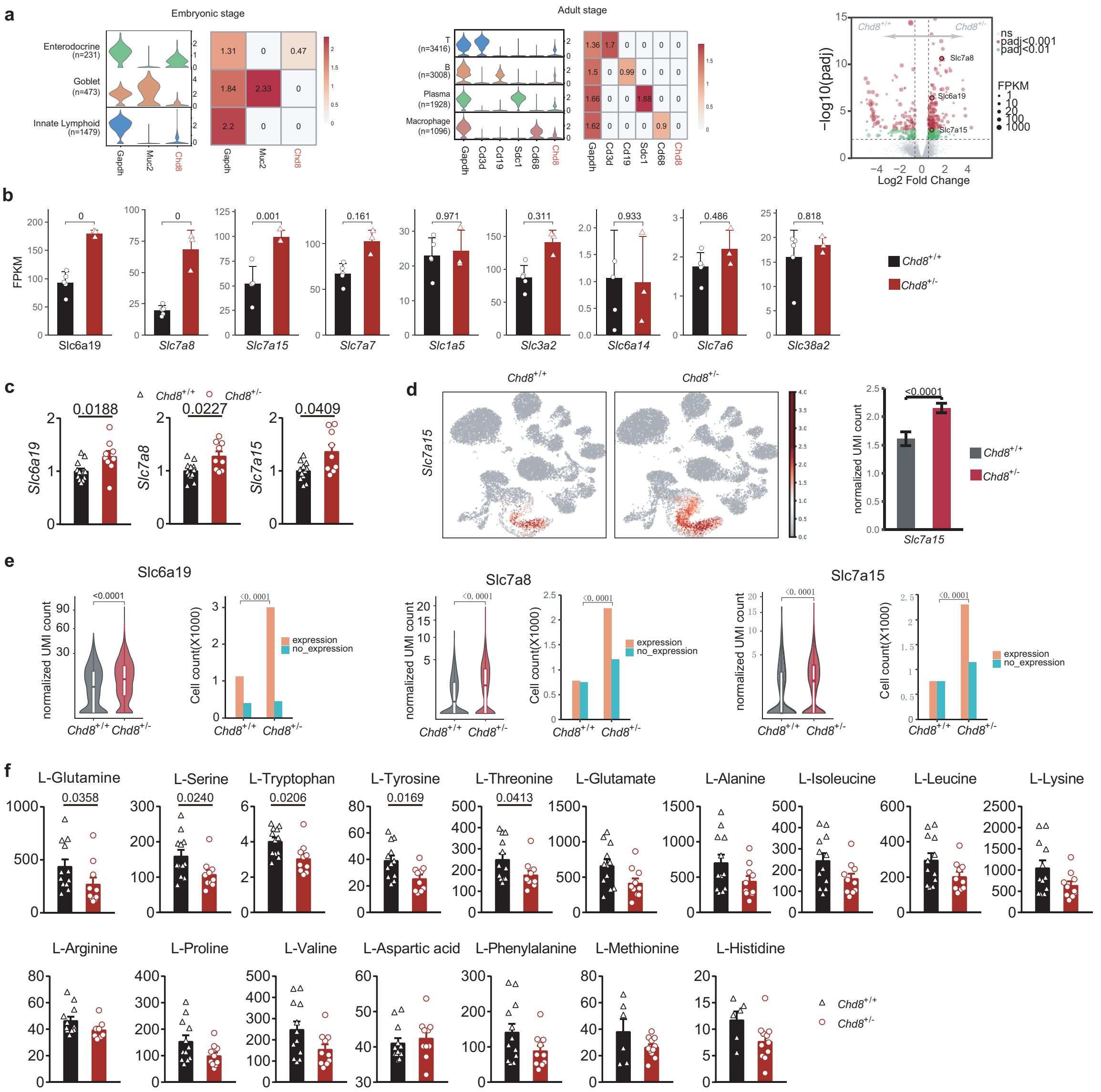

Supplementary Fig. 3 | The E/I imbalance in the *Chd8*$^{+/-}$ mice is involved in abnormalities in the intestine. a, Left and middle panels: The expression of *Chd8*, marker genes, and the housekeeping gene in immune cells at the embryonic (left panel) and adult stage (middle panel) from scRNA-seq; n indicates the numbers of cells. Right panel: Volcano plots comparing gene expression in the small intestines of the *Chd8*$^{+/+}$ and *Chd8*$^{+/-}$ mice. The intestinal amino acid transporters with significant differences between the *Chd8*$^{+/+}$ mice and the *Chd8*$^{+/-}$ mice are marked as black circles and indicated by name (right panel). DE genes using a strict threshold of adjusted $P < 0.01$ and $|\log2(cf)| > 0.585$ (right panel). **b**, Expression level of intestinal transporters responsible for L-glutamine transmembrane transport from intestinal RNA-seq data. **c**, qPCR analysis of *Slc6a19*, *Slc7a8*, and *Slc7a15* in the small intestine of 12-week-old mice. n = 12 and 9 mice, respectively. **d**, UMAP plots (left) and expression level (right) of *Slc7a15* in enterocytes from scRNA-seq. The bar plots represent the mean of abundance of *Slc7a15* in enterocyte cells (n=1527 and 3445 cells in *Chd8*$^{+/+}$ and *Chd8*$^{+/-}$ mice, respectively). Error bars

represent 95% CI of mean value. **e**, The expression level of *Slc6a19*, *Slc7a8*, and *Slc7a15* (violin plots) in enterocytes from scRNA-seq (first, third and fifth panels), and the numbers of cells expressing *Slc6a19*, *Slc7a8*, and *Slc7a15* (bar plots) in enterocytes from scRNA-seq (second, third and last panels). Box plots showed center line as median, box limits as upper and lower quartiles, whiskers as 1.5× interquartile range and dots as outliers. The *P* values in violin plots and bar plots were calculated by two-sided Wilcoxon rank sum test and two-sided Chi-squared test, respectively. **f**, Targeted metabolomics assays for amino acids in the feces of 8-week-old mice (μg/g). n = 12 and 10 mice, respectively. Source data are provided as a Source Data file. Quantitative data are shown as the mean ± SEM (**b**, **c** and **f**). Statistical analysis was determined by the two-tailed Mann-Whitney test (**c**, **d** and **f**).

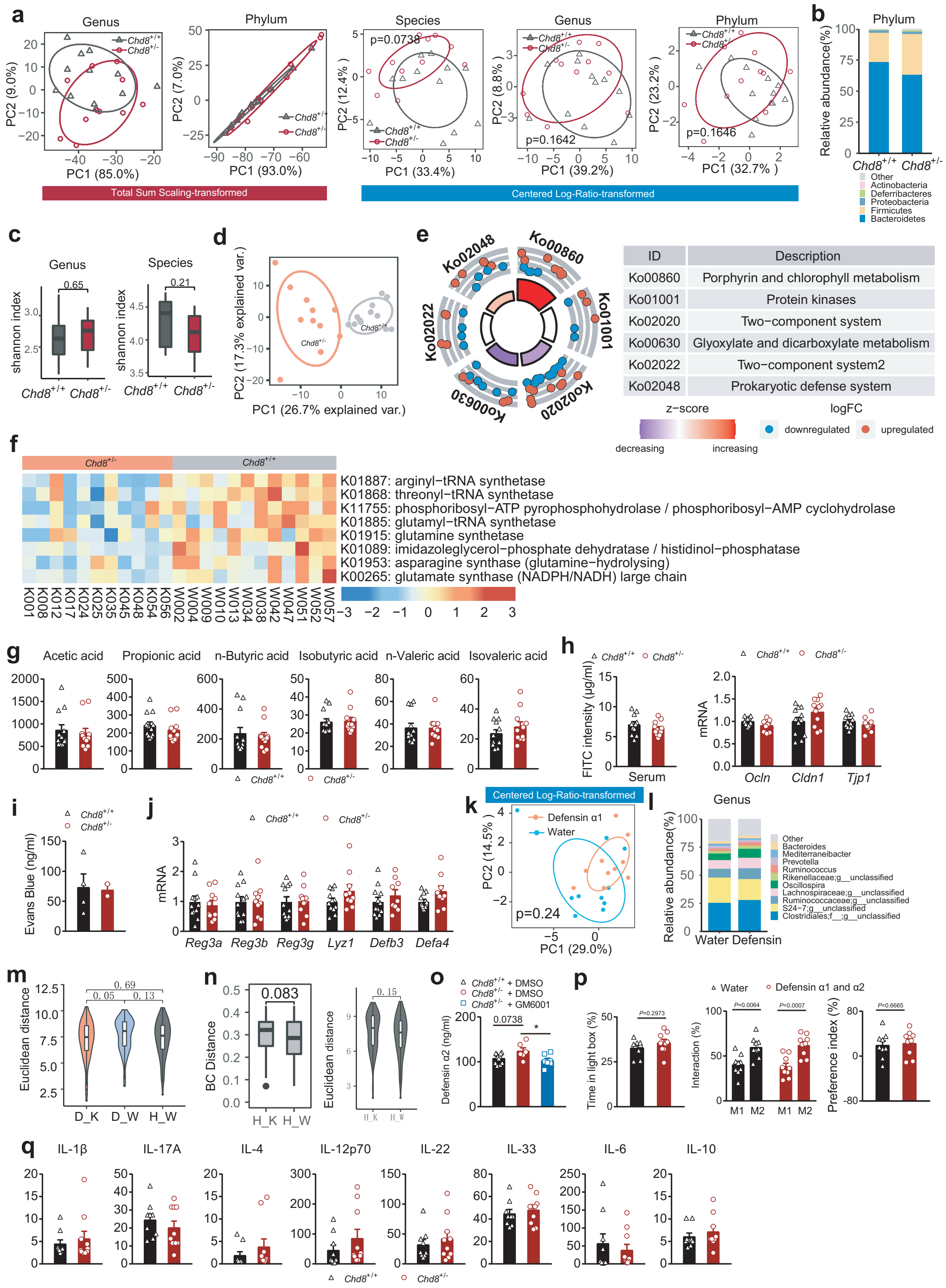

Supplementary Fig. 4 | Increased levels of alpha defensins contribute to gut microbiota dysbiosis in the *Chd8*$^{+/-}$ mice. a-f, Metagenomic sequencing analysis of the gut microbiota from 8-week-old mice. n=12 for *Chd8*$^{+/+}$ mice and n=11 for *Chd8*$^{+/-}$ mice. **a**, PCA of the microbiota composition at the genus and phylum levels (Total Sum Scaling-Transformed) (*P*=0.113 at the genus level; *P*=0.175 at the phylum level, PERMANOVA),

and PCA of the microbiota composition at the species, genus, and phylum levels (Centered Log-Ratio-Transformed). **b**, Phylum-level microbiota composition. **c**, Shannon index at the genus (left) and species (right) levels. Mann-Whitney test was used. **d**, PCA of 301 DE KOs (P<0.05, Mann-Whitney test) and significance (P=0.0001) determined by PERMANOVA. **e**, Enrichment analysis for DE KOs. A total of six KEGG pathways showed significant (P<0.05) differences. Red points indicate upregulation, and blue points indicate downregulation in the *Chd8*$^{+/}$ mice^{-}. The Z-score indicates a decrease (negative value) or increase (positive value) for the pathway. **f**, Heatmap of amino acid-related DE KOs in metagenomics (P<0.05, Mann-Whitney test). **g**, Targeted metabolomics assay for the SCFAs in the feces (μg/g) (n = 9 mice). **h**, FITC-dextran concentration in the serum and qPCR analysis of *Ocln*, *Cldn1*, and *Tjp1* in the small intestine of 12-week-old mice. n = 11 and 12 mice, respectively. **i**, Evans blue concentration in the cortex of 12-week-old mice. n = 4 and 2 mice, respectively. **j**, qPCR analysis of defensins in the small intestine of 12-week-old mice. n = 12 and 10 mice, respectively. **k-n**, A 16S rRNA sequencing analysis of gut microbiota from 63-day-old mice (n=10 mice). **k**, PCA of the microbiota composition at the genus level (Centered Log-Ratio-Transformed). **l**, Genus-level microbiota composition. **m**, Euclidean distance between D_W (defensin α1-treated mice and *Chd8*$^{+/+}$ mice), H_W (water-treated mice and *Chd8*$^{+/+}$ mice), and D_K (defensin α1-treated mice and *Chd8*$^{+/-}$ mice). The Box plots were based on 350 data points. **n**, BC distance and (left) Euclidean distance (right) between H_K (the water-treated mice and the *Chd8*$^{+/-}$ mice) and H_W (water-treated mice and *Chd8*$^{+/+}$ mice). The Mann-Whitney test was used to calculate the significance. The Box plots were based on 230 data points. **o**, ELISA analysis of the protein levels of defensin α2 in the cecal contents. n = 12, 7 and 7 mice, respectively. **p**, Behavioral tests of alpha defensin 1 and alpha defensin 2 gavaged mice (n=10 mice). Percentages of time spent in the light box in the light/dark box test (left panel). Percentages of interaction time (middle panel) and the preference index (right panel) in the social novelty preference session of the three-chamber social interaction test. **q**, Multiplexed determination of serum cytokine levels (pg/ml). n = 9 and 10 mice, respectively. Source data are provided as a Source Data file. Quantitative data are shown as the mean ± SEM. Statistical analysis was determined by the two-tailed Mann-Whitney test (**g**, **h** (left panel), **i, p** (left and right panels) and **q**), one-way ANOVA with Tukey's multiple comparison test (**o**), and two-way ANOVA with two-tailed Turkey's test for multiple comparisons (**h** (right panel), **j** and **p** (middle panel)). Significance was indicated by P value. Box plots (**m and n**) showed center line as median, box limits as upper and lower quartiles, whiskers as 1.5× interquartile range and dots as outliers. Ellipses were drawn around each group's centroid (95%) (**a, d and k**).

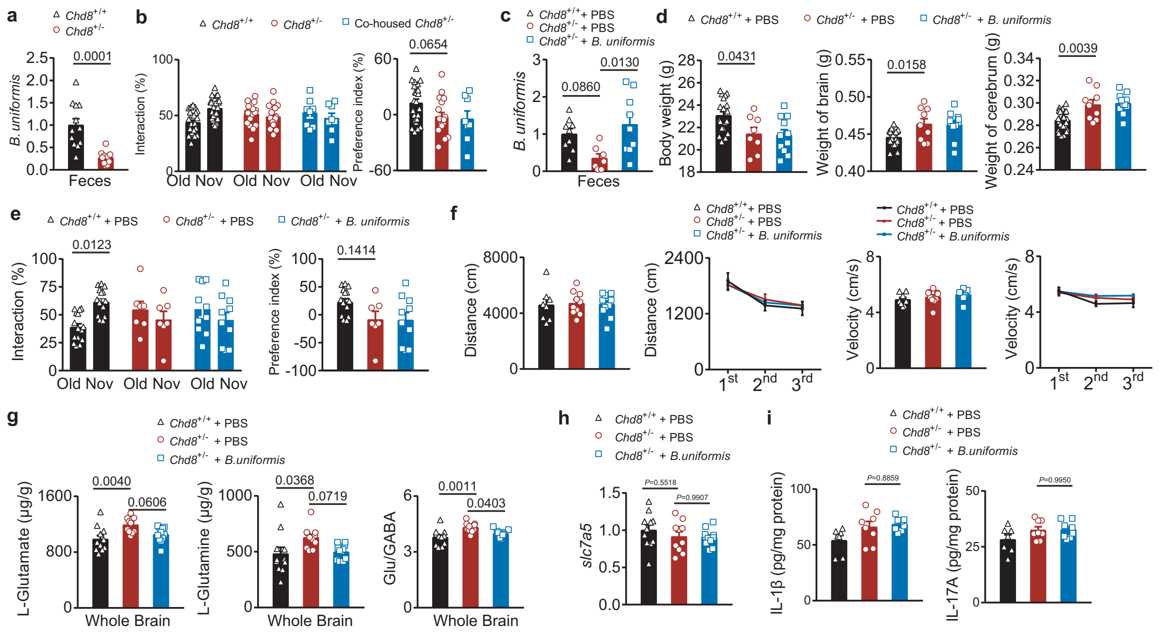

Supplementary Fig. 5 | *B. uniformis* improves the ASD-like behaviors and restores E/I imbalance in the *Chd8*$^{+/-}$ mice. a, qPCR analysis of *B. uniformis* in the feces from 8-week-old mice. n = 12 and 10 mice, respectively. **b**, Percentages of the interaction time (left) and the preference index (right) in the novel object recognition test. Nov, novel object; Old, old object. n = 24, 18 and 8 mice, respectively. **c**, qPCR analysis of *B. uniformis* in the feces from 8-week-old mice. n = 9, 7 and 9 mice, respectively. **d**, Body weight, weight of the whole brain and weight of the cerebrum of 12-week-old mice. n = 19, 8, 11, 23, 9, 11, 23, 9 and 11 mice, respectively. **e**, Percentages of the interaction time (left) and the preference index (right) in the novel object recognition test. Nov, novel object; Old, old object. n = 15, 7 and 10 mice, respectively. **f**, The total distance traveled (left two panels) and the velocity of movement (right two panels) in the open-field test across the total 30 minutes and every ten minutes. n = 8, 10 and 11 mice, respectively. **g**, Levels of L-glutamate, glutamate/GABA (Glu/GABA), and L-glutamine in the whole brain detected by targeted metabolomics assays (n=11 mice). **h**, qPCR analysis of *Slc7a5* in the brain. n = 11, 10 and 11 mice, respectively. **i**, ELISA analysis of cytokine levels in the intestine. n = 7, 8 and 8 mice, respectively. Source data are provided as a Source Data file. Quantitative data are shown as the mean ± SEM. Statistical analysis was determined by the two-tailed Mann-Whitney test (**a**), one-way ANOVA with two-tailed Tukey's multiple comparison test (**b** (right panel), **c**, **d**, **e** (right panel), **f** (first and third panel), **g**, **h** and **i**) and two-way ANOVA with two-tailed Turkey's test for multiple comparisons (**b** (left panel), **e** (left panel) and **f** (second and last panel)). Significance was indicated by *P* value.

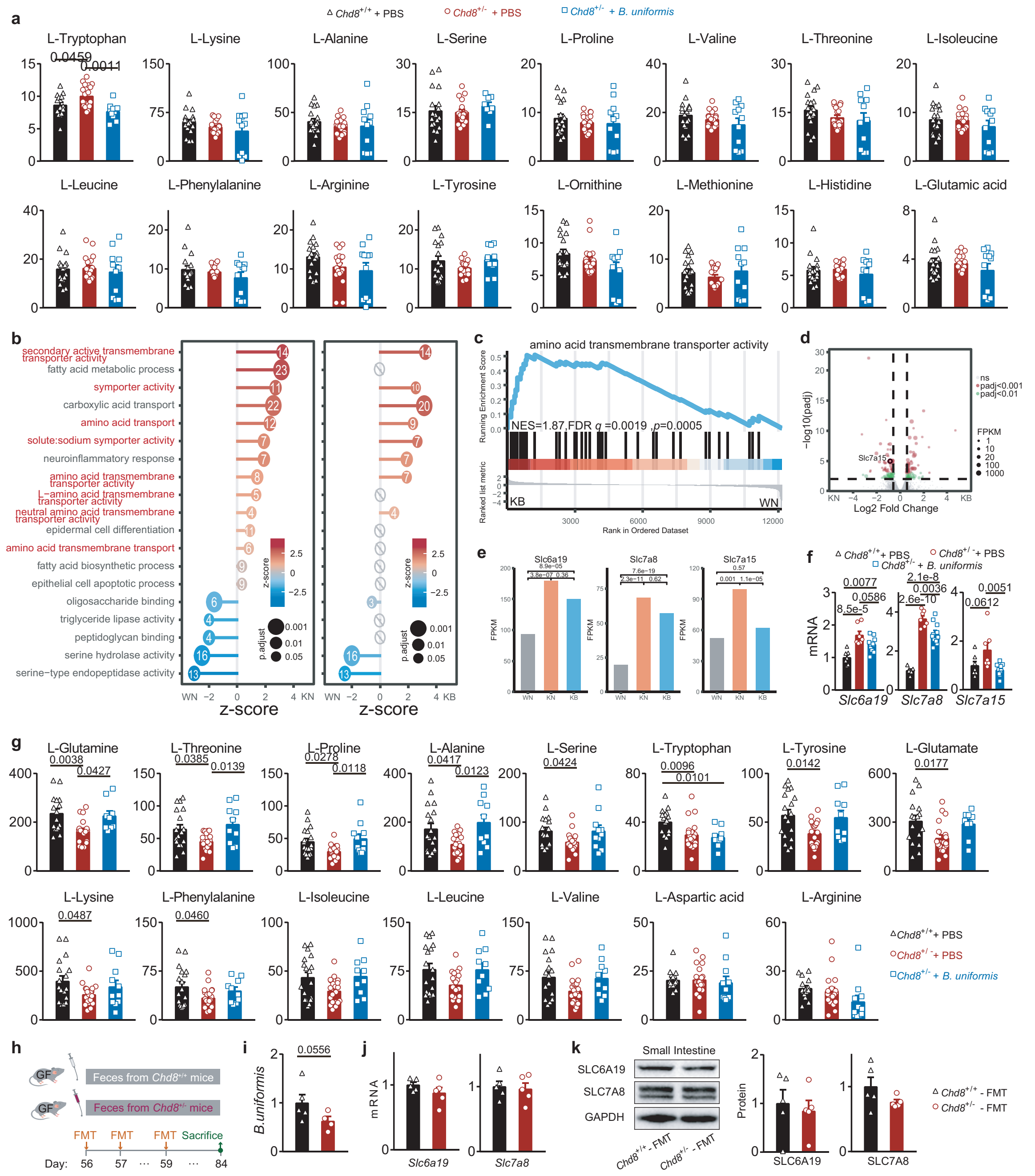

Supplementary Fig. 6 | *B. uniformis* decreases the expression of intestinal amino acid transporters in the *Chd8*$^{+/-}$ mice. a, Targeted metabolomics assays for amino acids in the serum of 12-week-old mice. n = 18, 18 and 10 mice, respectively. **b-e**, Bulk RNA-seq analysis of the small intestine of 12-week-old mice. There were n=3, n=3 and n=5 mice in the KN, KB and WN groups, respectively (WN: *Chd8*$^{+/+}$ mice gavaged with PBS; KN: *Chd8*$^{+/-}$ mice gavaged with PBS; KB: *Chd8*$^{+/-}$ mice gavaged with *B. uniformis*). **b**, Enrichment analyses for KEGG pathways of DE genes (adjusted P< 0.01 and |log2(cf)| > 0.585) between WN and KN (left) and WN and KB (right). The numbers in the circle indicate the count of DE genes in the pathways. **c**, A GSEA plot shows the enrichment of the amino acid transmembrane transporter activity pathway in small intestinal cells of KB

compared to WN. **d**, Volcano plots comparing gene expression in the small intestines of KN and KB. **e**, Gene expression analysis of *Slc6a19*, *Slc7a8*, and *Slc7a15*. The significance was determined by Deseq2, and the adjusted *P* is indicated in the picture. **f**, qPCR analysis of the *Slc6a19*, *Slc7a8*, and *Slc7a15* levels in the intestine of 12-week-old mice. n = 7, 7 and 11 mice, respectively. **g**, Targeted metabolomics assays for the amino acids in the feces of the mice (μg/g). n = 18, 21 and 11 mice, respectively. **h**, Schematic diagram for fecal microbiota transplantation (FMT). **i**, qPCR analysis of *B. uniformis* in the cecal content. n = 5 and 4 mice, respectively. **j**, qPCR analysis of the *Slc6a19* and *Slc7a8* levels in the small intestine (n=5 mice). **k**, Western blotting analysis of the SLC6A19 and SLC7A8 protein levels in the small intestine (n=5 mice). Source data are provided as a Source Data file. Quantitative data are shown as the mean ± SEM. Statistical analysis was determined by the two-tailed Mann-Whitney test (**i** (one-tailed p value), **j** and **k**), one-way ANOVA with two-tailed Tukey's multiple comparison test (**a**, **f** and **g**). Significance was indicated by *P* value.

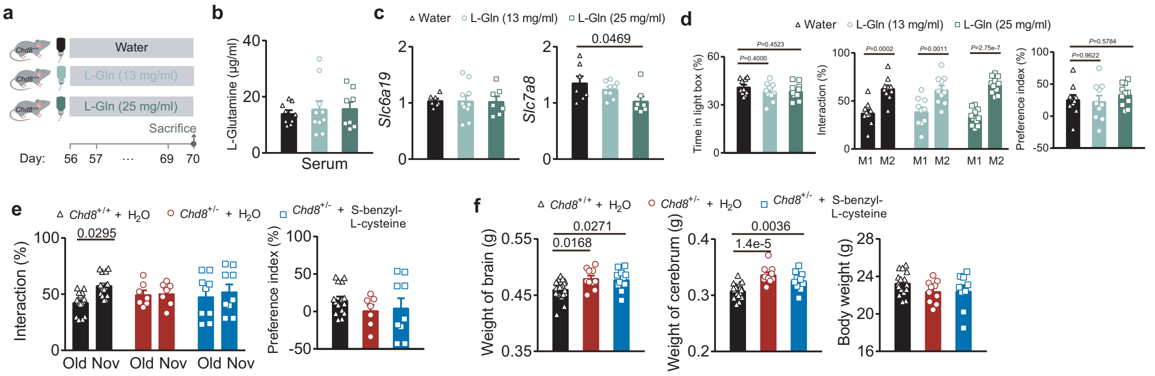

Supplementary Fig. 7 | S-benzyl-L-cysteine improves the ASD-like behaviors and E/I imbalance in the *Chd8*$^{+/-}$ mice. a, Schematic diagram of L-glutamine supplementation in the drinking water of 8-week-old *Chd8*$^{+/+}$ mice for 14 days. **b**, The level of L-glutamine in the serum. n = 9, 10 and 8 mice, respectively. **c**, qPCR analysis of the *Slc6a19* and *Slc7a8* levels in the small intestine. n = 7, 9 and 7 mice, respectively. **d**, Behavioral tests of glutamine-treated mice. n = 10, 10 and 11 mice, respectively. Percentages of time spent in the light box in the light/dark box test (left panel). Percentages of interaction time (middle panel) and the preference index (right panel) in the social novelty preference session of the three-chamber social interaction test. **e**, Percentages of the interaction time (left) and the preference index (right) in the novel object recognition test. Nov, novel object; Old, old object. n = 16, 7 and 9 mice, respectively. **f**, Body weight, weight of the whole brain, and weight of cerebrum of 12-week-old mice. n = 21, 10, 11, 21, 10, 11, 16, 10 and 9 mice, respectively. Source data are provided as a Source Data file. Quantitative data are shown as the mean ± SEM. Statistical analysis was determined by one-way ANOVA with two-tailed Tukey's multiple comparison test (**b**, **c**, **d** (left and right panels), **e** (right panel) and **f**) and two-way ANOVA with two-tailed Turkey's test for multiple comparisons (**d** (middle panel) and **e** (left panel)). Significance was indicated by *P* value.

Supplementary Table 1. Primers used in this study.

Sequences of primers	
Primers	Sequences (5' to 3')
Chd8-exon1-R1	TGACTTTTCCACCGTTGGCTGT
Chd8-mut-F1/R1	TCATCAGCAAGTGACCAGAGGA / TGACTTTTCCACCGTTGGCT
Defa1-F/R	AGAGAACGCATGAATGGAACC / GAAGTGGTCATCAGGCACCA
Defa2-F/R	TCACCAATTCTCCAGGTGACT / CATCTGTGTTTTGGATAGGATCA
Defa4-F/R	GCCAAGAAGGGTCTGCTCT / ATTCCACAAGTCCCACGAA
Reg3a-F/R	GTCCTCAACAGTATTTCCTGGA / TCATAACTAAGGCATAGCAGTGG
Defb3-F/R	TTGGCAGTTGTGGAGTTCCT / GGAGGAGCAAATTCTGGTGT
Slc6a19-F/R	AAGCCTCAGCTGGAAAAATACTGC / TTGAGAAAGATACATTTTAGACTCAACAACC
Slc7a8-F/R	GGTCTCCCAACTTCCTACCTAGCT / GAGAGAGAGAGAGACAATAATTCAAGGAG
Slc7a15-F/R	TCAAAGCTGGATCCAAGGCAT / TACTGTTTATTGCACTGTTCAAGCAAA
Cldn1-F/R	GACTGTTGATGATGGTTATCGG / AGATGGTAAGGTACAGCCAAGG
Tjp1-F/R	TGGGCAAGGGATAGGAGTG / ATATGGCTGGCCAATCGA
Gapdh	TGACATCAAGAAGGTGGTGA / TCCACCACCCTGTTGCTGTA
