# Supplementary material for: Changes to gut amino acid transporters and microbiome associated with increased E/I ratio in Chd8+/− mouse model of ASD-like behavior
Source: Nat Commun. 2022 Mar 3;13:1151. doi: 10.1038/s41467-022-28746-2 (PMC8894489; doi:10.1038/s41467-022-28746-2)
Supplement: Supplementary file 2 — Reporting Summary [file 41467_2022_28746_MOESM2_ESM.pdf]

nature research

Corresponding author(s): Fangqing Zhao

Last updated by author(s): 2021/04/24

Reporting Summary

Nature Research wishes to improve the reproducibility of the work that we publish. This form provides structure for consistency and transparency in reporting. For further information on Nature Research policies, see our Editorial Policies and the Editorial Policy Checklist.

Statistics

For all statistical analyses, confirm that the following items are present in the figure legend, table legend, main text, or Methods section.

n/a	Confirmed	
☐	☒	The exact sample size (n) for each experimental group/condition, given as a discrete number and unit of measurement
☐	☒	A statement on whether measurements were taken from distinct samples or whether the same sample was measured repeatedly
☐	☒	The statistical test(s) used AND whether they are one- or two-sided<br>*Only common tests should be described solely by name; describe more complex techniques in the Methods section.*
☐	☒	A description of all covariates tested
☐	☒	A description of any assumptions or corrections, such as tests of normality and adjustment for multiple comparisons
☐	☒	A full description of the statistical parameters including central tendency (e.g. means) or other basic estimates (e.g. regression coefficient) AND variation (e.g. standard deviation) or associated estimates of uncertainty (e.g. confidence intervals)
☐	☒	For null hypothesis testing, the test statistic (e.g. F, t, r) with confidence intervals, effect sizes, degrees of freedom and P value noted<br>*Give P values as exact values whenever suitable.*
☒	☐	For Bayesian analysis, information on the choice of priors and Markov chain Monte Carlo settings
☒	☐	For hierarchical and complex designs, identification of the appropriate level for tests and full reporting of outcomes
☒	☐	Estimates of effect sizes (e.g. Cohen's d, Pearson's r), indicating how they were calculated

Our web collection on statistics for biologists contains articles on many of the points above.

Software and code

Policy information about availability of computer code

Data collection	All software used for data collection is commercially available.<br>Open-field test: LabState software (AniLab Scientific Instruments Co., Ltd.)<br>mEPSCs and mIPSCs:PCLAMP 10.6 software (Molecular Devices)
Data analysis	All software used for data analysis is commercially available.<br>Immunofluorescence and Western blot: Image J (64 bit), Photoshop CS5 (Adobe)<br>Bulk RNA-seq: HISAT2 (version 2.0.5)-StringTie (version 1.3.4) pipeline, DESeq2 (version 1.24.0), clusterProfiler (version 3.12.0)<br>scRNA-seq: Cell Ranger (version 3.1.0), Scanpy (version 1.6.0), Scran (v1.16.0), Harmony (version 0.0.5), UMAP, (version 0.4.6)<br>Taxonomic analysis: cutadapt (version 1.14), BowTie2 (version 2.3.2), DIAMOND (version 0.9.24), QIIME2, vegan (version 2.5.6), ggbioplot (version 0.55)<br>Functional analysis of metagenomic data: HUMAnN2 (version 0.11.1), clusterProfiler, GOplot (version 1.0.2)<br>Statistics analysis: GraphPad Prism 2.0 (GraphPad)

For manuscripts utilizing custom algorithms or software that are central to the research but not yet described in published literature, software must be made available to editors and reviewers. We strongly encourage code deposition in a community repository (e.g. GitHub). See the Nature Research guidelines for submitting code & software for further information.

Data

Policy information about availability of data

All manuscripts must include a data availability statement. This statement should provide the following information, where applicable:

- Accession codes, unique identifiers, or web links for publicly available datasets
- A list of figures that have associated raw data
- A description of any restrictions on data availability

The authors declare that all data supporting the findings of this study are available within the paper and its supplementary information files.

Field-specific reporting

Please select the one below that is the best fit for your research. If you are not sure, read the appropriate sections before making your selection.

[x] Life sciences [ ] Behavioural & social sciences [ ] Ecological, evolutionary & environmental sciences

For a reference copy of the document with all sections, see nature.com/documents/nr-reporting-summary-flat.pdf

Life sciences study design

All studies must disclose on these points even when the disclosure is negative.

Sample size	Sample sizes for all data sets were determined based on pilot data or previously published data applicable to the specific experiment with similar methodologies (Platt, R. J. et al., 2017 (https://doi.org/10.1016/j.celrep.2017.03.052); Buffington, S. A. et al.,2016 (https://doi.org/10.1016/j.cell.2016.06.001); Sharon, G. et al., 2019 (https://doi.org/10.1016/j.cell.2019.05.004)). Sample sizes are reported in Figure legends or Figures.
Data exclusions	No data were excluded from the analyses.
Replication	We define each mouse as comprising an independent experiment. At least three mice comprised each experimental group. Each data set is a combination of at least two successful experimental replications. All attempts were successful.
Randomization	CHD8 haploinsufficiency mice and their wild type littermates used in this study were randomly allocated into various experimental groups.
Blinding	Data analysis was performed by a blinded researcher to the experimental groups. For all experiments, the individual that performed the experiments was independent of the individual that analyzed the data.

Reporting for specific materials, systems and methods

We require information from authors about some types of materials, experimental systems and methods used in many studies. Here, indicate whether each material, system or method listed is relevant to your study. If you are not sure if a list item applies to your research, read the appropriate section before selecting a response.

Materials & experimental systems

n/a	Involved in the study
[ ]	[x] Antibodies
[x]	[ ] Eukaryotic cell lines
[x]	[ ] Palaeontology and archaeology
[ ]	[x] Animals and other organisms
[x]	[ ] Human research participants
[x]	[ ] Clinical data
[x]	[ ] Dual use research of concern

Methods

n/a	Involved in the study
[x]	[ ] ChIP-seq
[x]	[ ] Flow cytometry
[x]	[ ] MRI-based neuroimaging

Antibodies

Antibodies used	Primary antibodies: CHD8 (1:1000, ab114126, Abcam), HRP-conjugated anti-GAPDH (1:5000, BE0034, Easybio, Beijing, China), SLC6A19 (1:5000, ab180516, Abcam), SLC7A8 (1:5000, ab75610, Abcam), Oxytocin (1:2000, 20068, ImmunoStar).<br><br>Secondary antibodies: Goat anti-Rabbit IgG (H+L) Cross-Adsorbed Secondary Antibody (1:500, Alexa Fluor 594, A-11012, Thermo Fisher Scientific), Goat anti-Rabbit IgG(H+L)-HRP (1:5000, BE0101, Easybio, Beijing, China), Goat anti-Mouse IgG(H+L)-HRP (1:5000, BE0102, Easybio, Beijing, China)<br><br>Stains: DAPI (C1002, Beyotime, Haimen, China)

Validation	Validation statements for the commercial antibodies are available on the manufactures websites.<br><br>The primary antibodies were also validated in previous studies:<br>CHD8: Gompers, A. L. et al., 2017 (https://doi.org/10.1038/nn.4592)<br>Oxytocin: Buffington, S. A. et al.,2016 (https://doi.org/10.1016/j.cell.2016.06.001)<br>SLC6A19: Castilla-Madrigal, R. et al., 2019 (https://doi.org/10.1016/j.jnutbio.2019.108264)<br>SLC7A8: Gigena, N. et al., 2017 (10.1530/JOE-16-0423)

Animals and other organisms

Policy information about studies involving animals; ARRIVE guidelines recommended for reporting animal research

Laboratory animals	Wild type C57BL/6J mice were purchased from SPF (Beijing) Biotechnology Co., Ltd (Beijing,China). CHD8 haploinsufficiency mice (C57BL/6J background) were established by the Nanjing Biomedical Research Institute of Nanjing University (NBRI, China).<br>The mice were bred by crossing male heterozygous CHD8 haploinsufficiency with female wild-type mice (C57BL/6J). Mice that were crossed for more than three generations were used for experiments. The mice used in this study were at the age of 8-14 weeks. Unless stated otherwise, male mice or samples from male mice were used in the experiments of this study.
Wild animals	No wild animals were used in this study.
Field-collected samples	No field-collected samples were used in this study.
Ethics oversight	All animal experiments were performed in accordance with the National Institute of Health Guide for the Care and Use of Laboratory Animals. All procedures were approved by the Animal Ethics Committee at the Institute of Zoology, Chinese Academy of Sciences (IOZ-IACUC-2020-034).

Note that full information on the approval of the study protocol must also be provided in the manuscript.
